# Interaction in acting training and its different manifestations in novice and professional actors

**DOI:** 10.3389/fpsyg.2022.949209

**Published:** 2023-01-09

**Authors:** Jingyan Sun, Takeshi Okada

**Affiliations:** ^1^Department of Interdisciplinary Information Studies, The University of Tokyo, Tokyo, Japan; ^2^Department of Educational Psychology, The University of Tokyo, Tokyo, Japan

**Keywords:** acting, interaction, theatrical performance, utterance analysis, expertise, social understanding

## Abstract

This study aimed to identify the characteristics of interactions during acting training and the underlying intrapersonal changes evoked by a training process that emphasizes paying attention to a partner (the Meisner technique). This was operationalized by conducting a *post-hoc* analysis and categorizing the utterances made by novice and professional actors during acting training based on video and audio recordings. In Study 1, novice participants tended to change their way of communication as the course progressed, decreasing the number of utterances that simply described the partner’s behavior and increasing those that speculated about the partner’s inner state. We then used a different focus placed on the interaction, as implied by the different kinds of utterances used, to describe the divergences between novice and professional actors regarding their interaction characteristics. In Study 2, results showed that while professional actors devoted themselves more to the connection with their partner and demonstrated more balanced communication, novice actors relied on general inference to speculate about others’ affective states. By comparing the characteristics of the utterances between novice and professional actors as they played different roles or made switches (i.e., changing from passive to active utterance in communication), this study suggests that an important impact of acting training on social abilities relates to its potential to increase the levels of involvement in on-going interactions.

## 1. Introduction

Acting has the potential of being a promoter of social understanding and performance. Thus, acting techniques have been applied by scholars in interventions related to psychiatric treatment (Bailey, 2009; Tang et al., 2020) to reduce social deficiency (Chandler et al., 1974) and general education (Goldstein and Winner, 2012) to foster social abilities. Using validated psychological scales and measures, scientists and scholars have demonstrated that theater-based interventions improve social cognition and engagement in participants with autism spectrum disorder through interactive performance (Corbett et al., 2016, 2017; Ward et al., 2018) and facilitate the understanding of social issues in general education (Manzi et al., 2020; Massa et al., 2020). Indeed, a burgeoning number of scholars have been providing insights into the mechanisms underlying the simulation or experience of a character and its impact on social performance (McDonald et al., 2020). Two studies reviewing theories from both practitioners and psychologists suggest that acting methods designed to instruct actors on how to understand and play a role are parallel to theories on the role of empathy in social relationships (Verducci, 2000; Gallagher and Gallagher, 2019). Furthermore, empirical studies have suggested the potential effect of acting on social cognition through understanding and simulating a character; self-schemas are malleable for allowing the simulation of others to serve as a method for improving self-knowledge and facilitating social connections (Meyer et al., 2019), and the processes for such improvement and facilitation are related to the extent of the character’s similarity to the self and the extent of the simulation of the character’s mental state (Broom et al., 2021). Researchers studying the neural basis of the simulation of characters have also shown that the brain network recruited in social cognition tasks is similarly activated in simulation tasks (Tamir et al., 2016).

However, acting goes beyond understanding and simulating a character. Specifically, actual role-playing performance offers a vital context for people to be engaged in emotional experiences and social interactions as they happen (Tamir et al., 2016; Wilson-Mendenhall et al., 2019). Furthermore, the positive effect of acting in various aspects of social cognition stems not only from promoting the use and understanding of imagination but also from the actual performance of acting. For example, a study compared the pre- and post-test scores for psychological scales of an experimental group that performed acting tasks with the pre- and post-test scores of a control group that experienced only narrative reading; the researchers showed that acting was associated with improvements in several social abilities, such as empathy and emotional understanding (Watanabe and Kusumi, 2020). Upon measuring the effects of representations of violence on spectators and performers, another study showed stronger emotional responses in the latter than in the first (Berceanu et al., 2020). While examining the influence of acting experience on personality traits related to social abilities, as well as comparing the results for actors (exposed to different levels of acting training and embodied experience of theatrical performance) with those for the general population, academicians have found that professional actors have higher scores in extraversion, empathy, and theory of mind tasks than the general population and amateur actors (Nettle, 2006; Goldstein and Winner, 2010; Schmidt et al., 2021).

While these previous studies have identified the correlation between the embodied experience of acting (i.e., actual performance) and several social ability dimensions, the underlying mechanism of the positive influence of acting experience on social performance remains unclear (Panero, 2019). Even for the most studied dimension, empathy, convincing evidence that explains its causal link with acting remains absent (Winner, 2018), and the results of studies comparing the scores for empathy between groups of actors and non-actors do not consistently show higher scores among actors (Goldstein et al., 2009; Goldstein and Winner, 2012). Psychometric studies measuring performance in other dimensions of social abilities and social cognition further demonstrate that actors do not consistently show higher scores in these dimensions (Alfonso-Benlliure, 2021; Schmidt et al., 2021). Considering these studies showing that more acting experience does not necessarily bring about better scores in social abilities or social cognition, researchers have faced hardships in explaining the effect of the actual experience of acting. Furthermore, because these past studies have used self-reported materials for assessing social performance, they naturally neglect the unconscious part of social performance, and cannot assess the detailed process of related changes in participants (Gentzler et al., 2020; Panero and Winner, 2020). Considering this context, studies examining the details of the acting performance (e.g., behavioral characteristics, interactive changes, etc.) in-depth may offer valuable potential explanations for the inconsistent results regarding the social abilities of actors.

Studies that have examined vocal parameters during character portrayal show that trained actors have a more expansive repertoire of prosodic expression than novices (Berry and Brown, 2019; Matharu et al., 2022), facilitating their characterization of various types of roles with comprehensiveness and fluency. However, for actors to more clearly grasp the situation and emotional experience required of them in their acting work, it is important that they are competent not only in individual role-making and performance but also in stepping into the imaginary situation and interacting with other characters while playing the role. Research shows that the joint understanding of dramatic settings and their embedded interpersonal relationships emerge from actors experiencing dynamic interactions in training, rehearsal, or actual performance (Göthberg et al., 2018). Following these descriptions, this study focuses on interactions in acting, tries to identify their characteristics, and explores the influence of interactive role-playing experience on social understanding and intrapersonal changes.

Although limited, various studies addressing interactions in acting groups have attempted to clarify how collective interaction expands the possibility of improvisational theater (Sawyer, 2003; Sawyer, 2010) and contributes to the achievement of a balanced timing of utterances in rehearsal (Goan and Tsujita, 2007); these studies have yielded enlightening evidence. However, to understand the mechanisms that produce natural acting, we see the need for advancing the examinations in these studies by investigating the details of the behavioral changes of individual actors evoked by acting interactions; these investigations may yield relevant data upon which researchers can speculate on the intrapersonal changes that the actors experience in the process of engaging in acting interactions. Scientists in the social psychology and cognitive science fields have examined different dimensions of real-life interactions and discussed the mechanisms by which people act and feel when engaging in social interactions. They explain the following: social cognition is realized by reading the verbal and non-verbal information (i.e., social signals) in interactions (Pentland, 2010; Honda et al., 2016); social cognition results in social behavior adaptation (Clark, 1996; Zaki and Ochsner, 2011); people tend to try and infer others’ affective states while interacting (Ellsworth and Scherer, 2003; Wu et al., 2018; Ong et al., 2019), and people take actions considered to be appropriate to the specific context (de Melo et al., 2014; Lerner et al., 2015).

Concerning acting interactions, many stakeholders, for example, acting practitioners who advocate naturalistic acting (Kissel, 2000; Stanislavski, 2008; Meisner and Longwell, 2012)—highlight that the actor’s devotion to the situation on stage and effective communication with others on stage should resemble real-life situations, as this is vital for actors to experience how the character thinks and feels. Such an opinion is consistent with that of research on creativity that proposes the synergistic effect of elements (i.e., creators, action, the material, and social world, etc.) in a creative process (Glăveanu, 2013), which, in turn, sheds light on the existing tensions related to acting on stage (e.g., between an actor and the surrounding actors, between an actor and the environment, etc.) and how these tensions give rise to the actor’s creativity. Furthermore, acting performance can vary greatly, and this variation can often stem from the divergent interaction details in different acting stages; albeit, in text-based theater, this divergence is constricted to some extent by the play script (Goldstein and Levy, 2017). These different interaction details offer the possibility of breaking an acting performance down into measurable indicators and discussing the changes depending on the interaction between actors. That is, by examining acting interactions, researchers may be able to make inferences about intrapersonal changes. For example, Sun and Okada (2021) have examined the characteristics of utterances in acting training sessions that required actors to try to communicate with each other by describing their partner’s behavior under pre-set circumstances; by categorizing these utterances, the researchers found that utterance patterns varied by the actor’s role in a given acting context, which they then considered to be a manifestation of different attention distribution. These authors have also shown that during interactions, paying attention to one’s partner rather than to oneself encouraged actors to use various expressions and immerse themselves in the setting.

In the current study, the observations revolve around the Meisner technique (Meisner and Longwell, 2012), which is an acting training method that places the interaction between actors at the foundation of most of its practices in an attempt to stimulate actors’ response richness (further explanations are provided under “subsection 2.1”). Two fieldworks were conducted during acting courses delivered separately for novice and professional actors with the use of the Meisner technique. By using the utterance categorization proposed in the study by Sun and Okada (2021), the present study aims to examine the characteristics of novice actors’ utterances in acting training exercises, compare their utterances with those of professional actors engaged in comparable acting exercises, and reflect speculatively on the intrapersonal changes that may have been invoked by the observed acting interactions.

The study comprises two parts. Study 1 examines the characteristics of the acting interactions during the course for novice actors, focusing on the changes in the participants’ utterances and discussing how they relate to their intrapersonal changes. Study 2 investigates the differences between novice and professional actors in their utterance patterns and how they “switched” (the concept of “switch” is explained in “subsection 3.2.2.1”). This comparison is expected to provide preliminary explanations about: which part of the acting interaction evokes transformations vital for the authentic and naturalistic quality of performance pursued by the Meisner technique (which we explain below); how participants understand the social relationships they engage in during acting training; and how participants experience their affective changes within such relationships. Upon combining the two sets of results, we depict an important dimension of interaction in the specific setting of acting training and how utterances can be used when applying acting training methods to improve social abilities.

## 2. Materials and methods

We conducted fieldwork in two acting courses, one for novice actors (Field 1) and another for professional actors (Field 2), which used the Meisner technique as the training method. The instructor of both courses was Bobby Nakanishi, who had learned the Meisner technique in the United States and taken an active part in The Actors Studio, New York for 15 years. He has devoted himself to delivering acting instruction for actors in Japan since 2011 and started the acting course for university students in 2019. Course *****details are explored under “subsections 2.2 and 2.3” after an introduction of the Meisner technique practices in “subsection 2.1.” Then, the designs of Studies 1 and 2 are described under “subsections 2.4 and 2.5,” respectively.

### 2.1. Practices in the Meisner technique

The Meisner technique is a seminal training system aimed at achieving authentic acting (i.e., acting that feels like real-life behavior), and it has been widely used in the United States since the mid-20th century. It emphasizes paying attention to others and forging a real-time relationship during acting interactions (Meisner and Longwell, 2012). All training sessions in the selected fieldworks were performed using pairs. The most basic practice in this technique is called *Repetition*, requiring participants to formulate sentences about their partner’s behavior and repeat them. All other practices are based on the premises of this *Repetition* practice, albeit with the addition of various characters and situations. The sessions were designed to cultivate the actor’s sensitivity toward their partner and the context, thus helping participants to “live truthfully under imaginary circumstances” (Meisner and Longwell, 2012).

#### 2.1.1. Practice of *Repetition*

The participants began by staring at each other. They were required to pay full attention to their partner’s behavior and to form sentences describing what the partner was doing; there was only one rule: they should talk only about the partner, not about themselves, and should be concerned about the mutual relationship. The sentences were initiated by “You are …” and were followed by a predicate.

Any participant in a pair could start the session if one perceived something in their partner about which to formulate a sentence. After hearing the partner uttering something like “You are (predicate A; e.g., laughing),” one should repeat the sentence with the subject substituted by “I” [i.e., the reply should be as follows, “I am (predicate A).”]. The pair continued repeating the sentence with predicate A until one detected a change in the partner and formulated a new sentence. The new sentence should again be a description of the partner’s behavior [e.g., “You are (predicate B).”].

According to Meisner, the *Repetition* practice entails not a simple and naive replication of words, but a trigger for and a carrier of a real emotional experience. The training of *Repetition* is designed to lead participants to free themselves while communicating with their partner and to be honest to their inner impulse to respond more spontaneously. Meisner believed this to be an actor’s first step in the preparation for acting, making this practice the foundation of the Meisner technique.

#### 2.1.2. Advanced work based on *Repetition*

Meisner developed various training practices (hereinafter, advanced exercises) to allow for translating the authentic expressions evoked in *Repetition* to authentic performances on the stage. The different advanced exercises add different elements to *Repetition* to help actors adapt to various imaginary situations. Regardless of the added element, participants were required to communicate following the instructions for the *Repetition* practice, which, in turn, allowed them to draw upon their internal affection. This study analyzed training sessions related to two of such additional works.

In Field 1, novice actors experienced an advanced exercise called *Card* or *Puzzle*. In it, one participant (i.e., the executor) chooses to play with a card tower or a jigsaw puzzle (this choice is non-important for the task) and tries to finish it within 10 min. The executor also selects a reward for success and a punishment for failure, both of which serve as the motivation of the portrayed character to finish the specific task and to stimulate the participant. The other participant (i.e., the observer) observes the executor in silence for 2 or 3 min, which is a period that allows for the executor to focus on the task at hand (i.e., either the card tower or the jigsaw puzzle), then starts *Repetition* with the executor.

In Field 2, actors mainly practiced the advanced exercise called *Activity*. In it, an executor performs a 10-min task, with the content and the goal of this task being decided by the executor in advance. After the executor spends 2 or 3 min focusing on the task at hand, the observer starts *Repetition* with the executor.

### 2.2. Field 1: A one-semester course for novice actors

Field 1 comprised a 14-class acting course for university students (from April to July 2019) to experience the Meisner technique. The course began with an introduction to naturalistic acting and the Meisner technique, followed by the main part of the course where students could experience *Repetition*, *Card* or *Puzzle*, and other training methods. The last three classes of the course were acting practice classes, during which students tried to integrate what they had learned to analyze the script of a scene and perform it. The syllabus is listed in Table 1.

**Table 1:** Schedule of the acting course for novice actors.

Class no.	Contents
1–3	Self-introduction of participants, introduction to the course, warm-up
4–6	*Repetition* (data in Study 1)
7–8	*Card* or *Puzzle* (data in Study 2)
9–11	Group work based on *Repetition: As if, Animal, Gradation*
12–14	Scene work

#### 2.2.1. Participants

Sixteen undergraduate and graduate students from different departments joined the course, 12 of whom completed the course. Those who did not finish the course left at the stage of script analysis, the data of which were not analyzed; hence, we decided to use the demographic information of the 16 participants (which was collected at the end of the first class) for describing the sample. All participants (50% women, 50% men) were in their 20s, except for one person in the 60s, and only 13 participants specified their ages [mean (M) = 25 years; standard deviation (SD) = 10.7 years]. According to the demographic survey, no participant had received professional acting training.

Prior to the study onset, all participants were informed about the goal and the content of the research, as well as the commitment to data anonymization and protection. It was clarified that participation in the research was not related in any way to course credits, and that participants were free to withdraw from the study at any time. All 16 students provided their written consent to participate in the research.

#### 2.2.2. Data collection

All course classes, including training sessions and the related discussions, were recorded using a video camera and an audio recorder. Classes were held once a week for 14 weeks, each with a duration of 105 min. Participants performed a total of 22 *Repetition* sessions across three classes (from the fourth to the sixth class; Table 1). Regarding advanced exercises, *Card* or *Puzzle* was the most practiced (15 sessions across the seventh and the eighth classes). Students were also allowed to voluntarily submit a self-reflection sheet after each class, wherein they were instructed to write on the feelings, problems, and discoveries they experienced in that day’s practice.

### 2.3. Field 2: A one-term course for actors

Field 2 was a 15-class acting course for people who intend to work as professional actors (from April to August 2019). The course did not deliver the whole set of practices under the Meisner technique because these advanced participants already possessed fundamental Meisner training. Instead, this course was mainly comprised of *Activity* training sessions across the 15 classes. Nonetheless, the instructor occasionally assigned other advanced exercises to participants according to learning needs. For *Activity* sessions, before each class and after being present in the class site, participants decided on a task that they would perform when playing the role of executors. Then, the instructor arranged them in pairs for the practicing session to start.

#### 2.3.1. Participants

Field 2 comprised 20 actors (80% women, 20% men), and 16 specified their ages in the demographics survey (the remaining four preferred not to answer; *M* = 36 years; SD = 11.5 years). All participants worked as professional actors and 13 responded to an item on acting experience, with two reporting less than 1 year, four reporting 1–5 years, and seven reporting over 5 years of experience (*M* = 8 years; SD = 6.8 years). They also reported on their training time in the Meisner technique (*M* = 0.8 years, SD = 0.5 years).

All the participants were informed about the goal and contents of the research, that research participation was independent of the participation in the training, and that participants were free to withdraw from the study at any time during the course. All participants provided their written consent.

#### 2.3.2. Data collection

All course classes, including training sessions and the corresponding comments from the instructor, were recorded with a video camera and an audio recorder. Classes were held once a week for 15 weeks, each with a duration of 240 min, and there were *Activity* sessions every week (for a total of 83 sessions). One session was excluded from analysis because it was interrupted halfway through, but all other 82 *Activity* sessions were analyzed.

### 2.4. Study 1

#### 2.4.1. Data set

To identify the characteristics of the novice actors’ interactions and their potential intrapersonal changes as they took the first step to experience the Meisner technique, we focused on their utterances in the three *Repetition* classes from Field 1. By excluding participants who were not present for all *Repetition* classes, the utterances of ten participants were analyzed.

We also used qualitative data from the participants’ self-reflection sheets. However, only two participants completed all the self-reflection sheets for the *Repetition* classes; this low rate of completion for the sheets is possibly related to their submission being voluntary. The answers from these two participants were included as supplements to the analysis of the utterances.

#### 2.4.2. Data processing

By referencing the video and audio recordings, the utterances in the *Repetition* sessions were transcribed by the first author (in Japanese). The predicate of each utterance was recorded on its first appearance—the repeated ones were omitted—along with the participant who produced the sentence. Utterances were divided into five categories following the categories listed in Table 2, which are based on prior research (Sun and Okada, 2021). The categories indicate the extent to which the participant producing the sentence reads the partner, ranging from simply stating the partner’s external movements to describing the behavior from a subjective perspective. All utterances were exclusively allocated to one of the five categories.

**Table 2:** Categories of utterances.

Number	Category	Definition	Examples
1	Description	Ordinary, “doable” verbs describing the external behavior of the other actor	Laugh, get closer, speak louder
2	Feeling	Words expressing one’s feelings about the external behavior of the other actor	Take a sharp look, seem to give up, not in a hurry
3	Evaluation	Words evaluating the external state of the other actor or the progress of the task	Be calm, not work, laugh in a strange way
4	Speculation	Words indicating what is assumed to be the inner state of the other actor	Be glad, worry, feel frustrated
5	Exclamation	Words uttered unintentionally, not following the rule “only speak about the other actor”	Ah, oh my

Sun and Okada (2021).

An inter-rater reliability analysis with two raters (the first author and a graduate student, with the latter receiving prior explanations about the outline of the research and the utterance categories) was conducted for utterance categorization. The concordance rate was 89%, and the kappa coefficient was 0.86, showing an almost perfect strength of agreement (Landis and Koch, 1977). The number of utterances made by each participant for each category in each *Repetition* session was counted and recorded. The descriptive statistics for the utterances in each class are summarized in Table 3.

**Table 3:** Descriptive statistics for each kind of utterance made by novice actors in each *Repetition* session.

Category	1st class		2nd class		3rd class	
	Mean	SD	Mean	SD	Mean	SD
Description	11.1	4.93	6.0	3.56	6.6	2.07
Feeling	4.1	2.18	5.9	3.73	3.1	2.47
Evaluation	3.3	3.13	3.7	3.30	1.9	2.85
Speculation	4.7	5.01	8.8	5.51	9.3	5.14
Exclamation	0.1	0.32	0.1	0.32	0.1	0.32

SD, standard deviation.

To examine whether utterances in each category (categorical data) changed significantly across classes, non-parametric tests were conducted to compare the number of utterances in each category per session among the three *Repetition* classes. Given the small sample sizes inherent to this type of research and the non-normality of the data set, non-parametric testing was deemed appropriate as it makes no assumption of the underlying population. We tested whether the utterances in each category per session can be regarded as samples following the same distribution.

### 2.5. Study 2

#### 2.5.1. Data set

Study 2 served to provide comparison data for examining the differences between novice and professional actors regarding the interactions during acting training using the Meisner technique. Although the novice and professional actors did not perform any tasks that were identical (mostly due to the different objectives and designs of the two courses), for the comparison analyses, we focused on utterances in the *Card* or *Puzzle* sessions for novice actors (from Field 1; 15 sessions) and those in the *Activity* sessions for professional actors (from Field 2; 82 sessions).

We decided it appropriate to make comparisons using these two advanced exercises based on their similarities (see “subsection 2.1.2” for further details), in that both require an executor and observer to interact in an imaginary situation (i.e., a specific task and goal that are both defined by the executor) while making utterances following the requirements of the *Repetition* practice; both advanced works also comprised 10-min sessions. Furthermore, though different in the number of exercise sessions, we included all the sessions in the comparison because we regarded sessions practiced by different pairs as different samples when focusing on the behaviors embedded in interaction. Utterances were recorded along with the executor or observer who made the sentence.

#### 2.5.2. Data processing

The utterances were transcribed, categorized, and counted in the same way as described in “Section 2.4.2.” The descriptive statistics of the utterances are summarized in Table 4. The inter-rater concordance of categorization rate was 91%, and the kappa coefficient was 0.88, showing an almost perfect strength of agreement (Landis and Koch, 1977). For categorical data that do not have a normal distribution, non-parametric tests were conducted to examine whether there were significant differences between novice and professional actors regarding the number of utterances in each category per session (not in total).

**Table 4:** Descriptive statistics for each kind of utterance in each session for novice actors (*Card* or *Puzzle* session) and professional actors (*Activity* session).

Category	Executor				Observer				Difference (observer minus executor)			
	Actor		Novice		Actor		Novice		Actor		Novice	
	Mean	SD	Mean	SD	Mean	SD	Mean	SD	Mean	SD	Mean	SD
Description	7.44	4.51	4.73	3.26	9.33	5.22	10.60	6.05	1.89	6.59	5.87	5.54
Feeling	1.44	1.55	1.20	1.90	2.51	2.30	7.27	6.08	1.07	2.76	6.07	6.20
Evaluation	1.41	1.58	0.40	0.61	2.09	2.26	2.13	2.50	0.67	2.87	1.73	2.19
Speculation	3.02	2.57	1.60	1.31	4.18	3.05	13.87	5.51	1.16	3.76	12.27	5.99
Exclamation	2.61	2.65	1.87	2.00	1.20	2.10	0.87	1.20	−1.41	3.03	−1.00	2.67

SD, standard deviation.

## 3. Results

### 3.1. Study 1

Friedman tests were conducted for the number of utterances per session across the three *Repetition* classes for novice actors, and independently for each utterance category. In addition, the Wilcoxon signed-rank test was used for pairwise comparisons, with the Bonferroni method being used for multiple comparison correction. A significance level of 0.05 was adopted.

**Figure 1:**
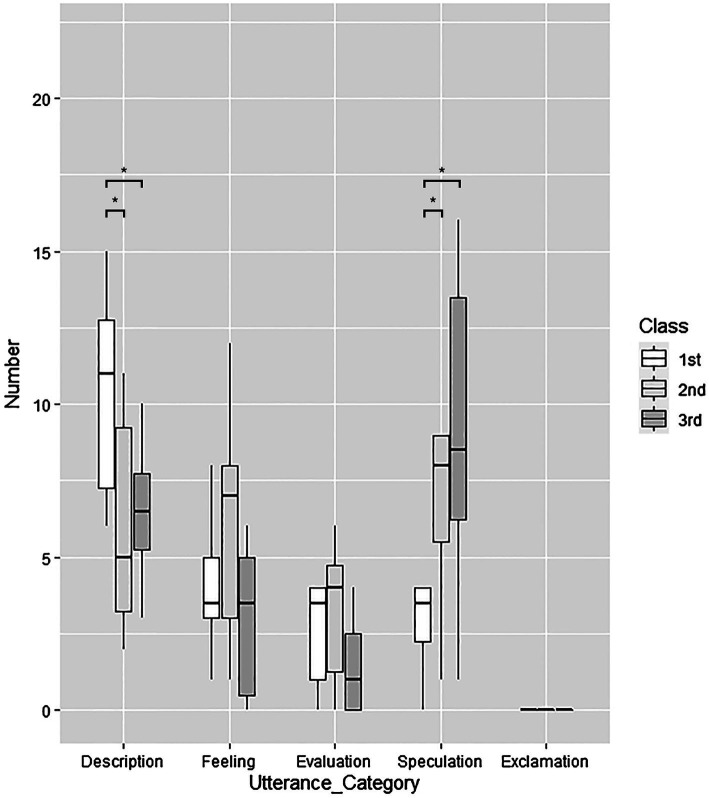
Novice actors’ changes with the progression of *Repetition* training in the number of each kind of utterance per session (^*^p < 0.05).

Results showed significant differences across the three classes for the number of Description utterances ($\chi^{2}$ = 11.56, *p* < 0.01). Pairwise comparisons showed that the Description utterances tended to decrease in number across the three classes (*p* = 0.01 for the comparison between the first and second classes; *p* = 0.02 for the comparison between the first and third classes). For the number of Speculation utterances, the differences across the three classes showed borderline statistical significance ($\chi^{2}$ = 5.4, *p* = 0.06). Unlike the Description utterances, Speculation utterances tended to become more frequent as time progressed (*p* = 0.02 for the comparison between the first and second classes; *p* = 0.04 for the comparison between the first and third classes; Figure 1). The differences across classes for the other utterance categories were all non-significant.

The findings show that the decrease in Description utterances and the increase in Speculation utterances occurred concomitantly, implying that participants attempted to form more sentences concerning the partner’s thoughts or feelings instead of simply describing their bodily behaviors. The Meisner technique requires one to try their best to focus on their partner’s behavior and understand the affective changes that occur during the scene as a natural result of their utterances, instead of focusing on the goal of their utterances. Still, novice actors seemingly found it difficult to avoid making simple changes to the sentence based on bodily behaviors and to change the sentence in a more complex manner to convey what their partner thought or felt. This may be because humans tend to make inferences about others’ affective conditions based on the perception and appraisal of actions and outcomes (Ong et al., 2019).

Despite the limited qualitative data (see “Section 2.4.1”), the comments in the self-reflection sheets of two participants supported the results from the analysis of utterance changes. In the first class, they were able to “pay attention to the partner,” and talked about how they felt in the interaction by describing their partner’s behavior. However, in the latter two classes, they reported being more inclined to “try to interpret what the partner is thinking” rather than to interpret the behavior itself, although a participant described ending up “narrowing down my sight” and “finding it difficult to interact in a natural way” upon trying to interpret the other’s thoughts. The consistency between the utterance analysis and these subjective reports shows that the novice actors faced some issues while attempting to focus on the partner’s thoughts and feelings, as well as demonstrates how their attempts at focusing on their partner significantly influenced their interactions with each other.

### 3.2. Study 2

#### 3.2.1. Comparison between novice and professional actors in the characteristics of utterances

As shown in Table 4, both professional and novice actors show similar interaction patterns, with observers producing more utterances of most categories than executors (per session). Non-parametric tests proved this observation to be true by comparing the number of utterances for each category between executors and observers across the two actor groups (professional actors: *p* < 0.05 for Description, Evaluation, and Speculation utterances, and *p* < 0.01 for Feeling and Exclamation utterances; novice actors: *p* < 0.01 for all categories, except for Exclamation, which showed a *p* > 0.05). This may be influenced by the differences between executors and observers in attention distribution patterns; particularly, while observers only need to focus on their partner’s behavior, executors must spend part of their energy on the task to attain the established goal, increasing their cognitive load and making interactive communication more difficult for them.

Another non-parametric test was run to compare the differences between professional and novice actors regarding the superiority in the number of utterances made by observers per session (i.e., calculated by taking the number of utterances of observers minus that of executors; Table 4). Results show that the superiority in the number of utterances by observers was more prominent in novice actors than in professional actors (*p* < 0.05 for Description utterances; *p* < 0.01 for Feeling and Speculation utterances). That is, the professional actor pairs seemingly kept a more balanced interaction (i.e., executors got more actively involved in the communication despite being occupied with another task) than the novice actor pairs. The disparity between these two groups provides a hint as to how close each group is to the core proposition of the Meisner technique, in that actors should attach importance to the interactions.

**Figure 2:**
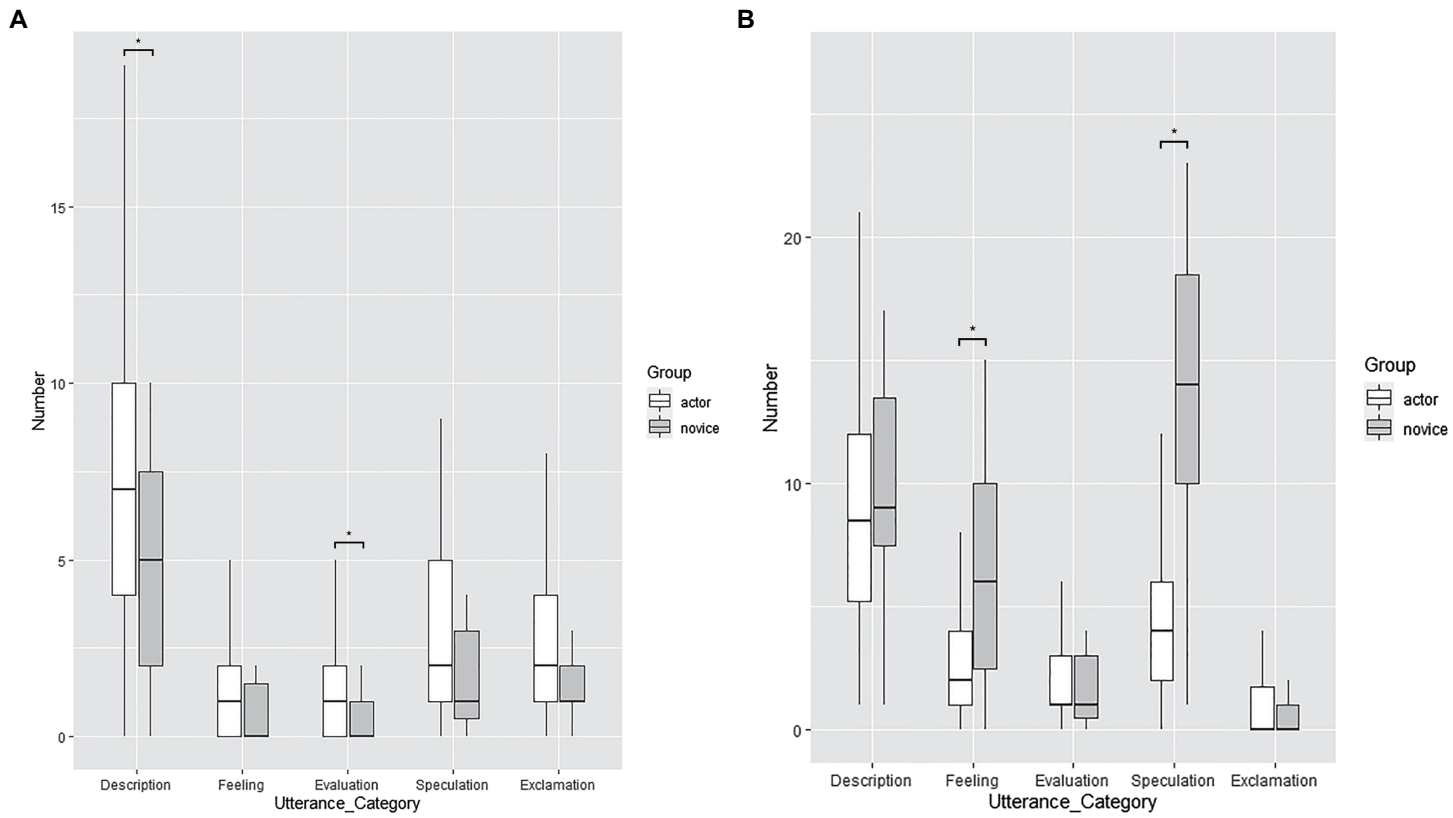
Difference between professional and novice actors in each kind of utterance per session (^*^p < 0.05), respectively for executors **(A)** and observers **(B)**.

To further understand how professional actors differ from novice actors, non-parametric tests were conducted to compare the number of utterances in each category for executors and observers per session between the two actor groups (Figure 2). Results show that the professional actor executors made significantly more Description (*p* = 0.046) and Evaluation utterances (*p* = 0.010) than the novice actor executors. The increased use of these two categories of utterances depicts that professional actor executors tended to describe or assess how their partner behaves and alters their behavior given the circumstances, something that can only be done by concomitantly focusing on the on-going interaction and the executor’s task. Meanwhile, the novice actor observers made significantly more Feeling (*p* = 0.001) and Speculation utterances (*p* < 0.001) than the professional actor observers. The greater use of these two categories of utterances shows that the novice actor observers tended to make judgments about their partner’s state. The findings also demonstrate that novice actors lacked variety in the details of their utterances, potentially showing their reliance on common expressions to make judgments instead of making real-time correlations between their partner’s state and the on-going situation in the scene. These results imply that professional and novice actors are likely to differ in the behavioral element they focus on when interacting with a partner.

#### 3.2.2. Difference between novice and professional actors in how they switch

##### 3.2.2.1. Identifying the point where the actor formulating a sentence “switches”

As described, the *Repetition* practice entails that any actor in a pair can form a new sentence at any time if the participant notices some change in the partner. This section is dedicated to analyzing the time points at which the speaker introducing a new predicate alternates in an actor pair, namely, when a “switch” occurs. For example, if an observer formulates a new sentence at a moment when the pair had been repeating a predicate that had been initiated by the executor, this is counted as a “switch.”

For the actor making the switch, the preceding sentence is about oneself, so it must be passively repeated, while the successive sentence after the switch will be about the partner, so it must be actively uttered. These switches indicate that the actor is paying actual attention to the partner and can make a transition from a passive to an active interaction, making the identification of the characteristics of switching and the examination of the differences between professional and novice actors regarding switching worthwhile investigations for this study.

##### 3.2.2.2. Differences between professional and novice actors regarding how they switch

The results of a preparatory non-parametric test indicated that there was no significant difference in the overall number of switches in each session between professional actors (*M* = 14.24, SD = 7.43) and novice actors (*M* = 13.00, SD = 8.62). Based on the five categories of utterance used in this study, 25 types of switching pairs can occur, and they are represented by the combination of the category of the preceding and successive utterances (e.g., the switch from a Description utterance to another Description utterance is represented as a [Description, Description], or a switching pair). We counted the number of times that each kind of the 25 types of switching pairs occurred. The descriptive statistics for number of switches is demonstrated in Table 5.

**Table 5:** Descriptive statistics for each kind of switch in each session for novice actors (*Card* or *Puzzle* session) and professional actors (*Activity* session).

Category	The utterance preceding the switch				The utterance succeeding the switch			
	Actor		Novice		Actor		Novice	
	Mean	SD	Mean	SD	Mean	SD	Mean	SD
Description	6.98	3.75	5.07	4.53	6.28	3.91	5.53	4.03
Feeling	1.38	1.46	1.80	2.14	1.72	1.51	1.67	1.59
Evaluation	1.23	1.25	0.67	0.90	1.44	1.47	0.67	1.11
Speculation	2.79	2.11	3.67	2.85	3.18	2.31	3.40	2.67
Exclamation	1.87	2.24	1.80	1.70	1.62	2.12	1.73	1.62

SD, standard deviation.

We also compared the number of switches per session with the same preceding utterance category between professional and novice actors; this served to examine whether there were differences by group in the time point when they made switches. The differences between groups nearly reached statistical significance only when the switch was preceded by a Description utterance (*p* = 0.054); when comparing the results for the switches with other preceding utterances between the professional and novice actors, the findings show very similar patterns, to the point where the results for the two different groups seemed to be from data of a single group.

Then, we compared the number of switches per session with the same successive utterance category by actor group; this served to examine the utterance category used to make the switch. Professional actors were significantly more likely than novice actors to switch using Evaluation utterances (*p* = 0.03), but no significant differences were found for any other switching pairs.

Overall, the professional and novice actors did not differ much in how they switched their utterances in the interactions. Still, the findings did show that professional actors had a slightly greater tendency than novice actors to be triggered to switch by Description utterances and to make the switch using Evaluation utterances. Both these utterances are associated with more attention paid to the on-going situation than to the partner’s subjective feelings, and professional actors seemed to perform better than novice actors in focusing on the on-going exchange. However, both actor groups showed the tendency to be triggered to switch by and to make the switch using Description utterances, followed by Speculation utterances (Table 5). A possible explanation for the predominance of Description utterances for both the triggering and performing of switches in both groups is that this category of utterances represents the simplest form of interaction. Meanwhile, the moderate rates for Speculation utterances in both the triggering and performing of switches in both groups may be associated with participants being generally apt to be touched by utterances relating to their internal states or attempting to use such utterances to bring about interaction changes.

## 4. Discussion

This study categorized the utterances made by novice and professional actors in acting training sessions (separate sessions for each actor group) that prioritized interaction to elicit authentic experiences. By doing so, it identifies the characteristics of the communication of novice actors under the framework of a specific acting training method (i.e., the Meisner technique) and shows how professional and novice actors differ in their communication characteristics. In Study 1, with the progression of the *Repetition* practices, novice actors tended to produce fewer Description and more Speculation utterances, showing an increasing inclination to make inferences about their partner’s inner states rather than about the external behavior. In Study 2, by comparing the data from professional and novice actors regarding their acting practices following pre-set circumstances (i.e., in each actor pair, an executor performed a task and an observer communicated with the executor following the rules of *Repetition*), novice actors manifested an interaction pattern that was less “balanced” than that of professional actors. Nonetheless, both actor groups showed similar tendencies regarding the quantity of utterances made by executors and observers, and this is possibly due to differences in acting role in attention distribution—executors have to shift their attention between a specific task and the communication with the observer, whereas observers need only communicate with executors, regardless of actor group.

Regarding utterance categories, professional actor executors produced more Description and Evaluation utterances than did novice actor executors, whereas novice actor observers produced more Feeling and Speculation utterances than did professional actor observers. These differences show that these two groups relate differently to the pre-set circumstances: professional actor executors seemingly paid more attention to the on-going interaction with observers, whereas novice actor observers leaned toward subjective communication (e.g., feelings). In addition, by looking into switches, both professional and novice actors showed a similar tendency to be triggered to switch by a preceding Description or Speculation utterance and to make the switch using these same two categories of utterances. However, professional actors performed significantly more switches that were triggered by a Description utterance and made using Evaluation utterances, showing a slightly different level of involvement in interaction between the two actor groups.

The integration of the results from Studies 1 and 2 enables this study to identify the characteristics and significance of the acting interactions in the process of reaching an authentic performance under imaginary circumstances; these circumstances were, in turn, put forward by the Meisner technique that attaches importance to focusing on the partner and devoting attention to interaction. Studies in cognitive science have described how personal efforts in character understanding and interpretation influence real performance (Noice and Noice, 2006; Ando, 2007). However, as a performing art, theater acting inevitably requires not only individual creation but also actual interaction among all the characters in the context (Glăveanu, 2013) to create an appealing reflection of real social relationships and life (Göthberg et al., 2018). The present study focuses on one aspect of such interaction within the context of a specific type of acting training, analyzing how participants use different utterances to describe the behavior of their partners (i.e., in actor pairs), which can then be used to infer how they relate to the on-going acting situation. The results partially reveal the difficulties that novice actors face when undergoing acting training following the Meisner technique, and how professional and novice actors place their focuses on different aspects of the on-going interactions. Researchers could further examine the relationship between attention distribution and interaction patterns in the future to enhance our knowledge of the underlying mechanisms of acting training exercises.

Furthermore, by comparing the interaction characteristics of novice actors with those of professional actors under the context of acting training using the Meisner technique, this study provides a new perspective on ways to measure the effect of interaction in role-playing. Specifically, this research shows that the actor’s level of involvement in interaction and attention switching fluency can be used to predict closeness to a natural performance. In our results, the pairs of professional actors (i.e., more experienced in acting training) adopted a more balanced communication than the pairs of novice actors, with professional actors being less restricted by the task of the character and showing greater success in keeping their attention on the interactions with their partner. These findings to some extent corroborate past research results that posit that through interactive training professional actors can perform smoothly as their respective characters and concentrate on the communication with each other simultaneously (Sun and Okada, 2021). Further, several possible explanations exist for the finding that professional actors showed an enhanced performance in the training sessions of our study; one would be their increased capacity for dual tasking, which is an executive function that has been shown to be improved by the practice of theater improvisation (Hansen et al., 2020). Another would be professional actors’ superior capacities for perceiving the actions required of them due to them being good task performers, which is a reflection of the mirror neuron theory suggesting a similar mechanism for both the observation and execution of actions (Calvo-Merino et al., 2005). Scholars may endeavor to further clarify the mechanisms behind this enhanced performance in the future.

Meanwhile, novice actors tend not to actively change the condition but try to read the partner in a way that resembles general inferences about others’ mental states (Thornton and Tamir, 2017), regardless of the particular context. According to Meisner (Meisner and Longwell, 2012), a better understanding of others is the natural result of this acting training method, not its goal; novice actors may face difficulties in adapting to this characteristic of the method. The present study highlights that it is important for actors to get involved in human interactions to improve their theatrical performances. That is, the process of acting training should not entail the simple replication of personalities and interpersonal relationships based on individual perspective-taking and understanding of a context, but rather encompass a convergent involvement in actual acting interactions to substantiate a realistic reflection of the social world.

By delivering evidence on the interaction and intrapersonal changes that professional and novice actors underwent through engaging in acting training using the Meisner technique, this study provides novel insights for research about creativity in acting and on general education. On the one hand, the detailed illustration of the processes in an acting training method that emphasizes interaction under imaginary circumstances takes an important step in explaining how actors become capable of connecting with their roles based on a script, thereafter enabling them to engage in various expressions depending on each performance. Despite the existence of various acting training methods, researchers have described that the scientific community has generally not attached importance to the value of these methods, nor have used valid frameworks for identifying the mechanisms of the effects of these methods on social abilities (Lippi et al., 2016). Addressing this shortfall, the current study is a preliminary step toward elucidating the actors’ transitions to fluent characterization through acting training. Researchers should conduct studies in the future that examine which instructions in the methods bring about these changes. On the other hand, by probing into the differences between professional and novice actors regarding their acting interactions, this study provides a new and inspiring method for scholars to use in future studies to achieve more accurate predictions of behavioral changes related to acting interventions. The Meisner technique emphasizes the actors’ involvement in real-time relationships and the experience based on such involvement. Accordingly, prior research has described that when the effect of interventions is assessed by scales for the general evaluation of social abilities, the results may be inconsistent (Goldstein et al., 2009; Goldstein and Winner, 2012). Thus, instead of using self-reported measures, this study collected and analyzed data related to the actor’s description of the behaviors of a partner and pertaining to the changes in these descriptions as the acting training progressed. This allowed us to measure the participants’ understanding of the acting situation and how involvement in the on-going situation relates to differences in interaction.

Furthermore, by attempting to elucidate the characteristics and effects of interaction embedded in acting training, this study further promotes a new approach that could be applicable to research in communication: using acting performances to explain the dynamics involved in human communication and the generation and inference of affective states within it. Indeed, prior researchers have emphasized that a focused examination of the details of acting may lead to the uncovering of the construction of smooth communication and social understanding (Greer, 2017; Nishiguchi et al., 2017; Valk et al., 2017). Acting training methods offer an environment with high ecological validity for research on communication, enabling researchers to extract multi-channel information in a controllable but natural communication setting, wherein participants undertake actions based on settings and relationships.

### 4.1. Limitation

Because of the class capacity the sample of the present study was small, which may hinder the statistical validity of the study results. Preliminary testing for normality led us to conduct the analysis based on non-parametric tests, which could be underpowered by the sample size. Furthermore, albeit that gender and age may have influenced the results related to the comparison analysis, the characteristics of the fieldwork and the small sample size did not allow us to control for differences in these two variables. Accordingly, it leads us to suggest that future studies verify the current findings with larger samples.

Additionally, considering the specificity of the acting training method used in the fieldworks, generalizations of the study results should be made with caution, and the transferability of the findings is limited. It should be noted that the discrepancy in the number of sessions each group was offered made it difficult to eliminate the effect of the accumulation of exercises from that of professional experience. As the Meisner technique course for professional actors was delivered according to each participant’s schedule, it was impossible to identify their first time attending the *Activity* session which is the same condition to that of novice actors. However, because this study focuses on the characteristics of their interaction, comparing the number of utterances or switches by category per session in these data sets is a viable method to capture the difference between professional and novice actors.

In addition, our fieldwork did not encompass thorough examinations of all the channels of interaction and did not provide a sophisticated explanation for the mechanisms underlying the effect of interactions on intrapersonal changes. Future research should focus on clarifying how multi-channel signals influence each other in acting and how they are related to fluency in role-playing and the experience of authentic emotions.

## Data availability statement

The original contributions presented in the study are included in the article/supplementary material, further inquiries can be directed to the corresponding author.

## Ethics statement

Ethical review and approval was not required for the study on human participants in accordance with the local legislation and institutional requirements. The patients/participants provided their written informed consent to participate in this study.

## Author contributions

JS performed the fieldwork, analyzed the data, and drafted the manuscript. TO provided critical revisions. All authors contributed to the article and approved the submitted version.

## Funding

This work was supported by the “Fostering Advanced Human Resources to Lead Green Transformation (GX)” project as a program of Support for Pioneering Research Initiated by the Next Generation (SPRING) of the Japan Science and Technology Agency, and by the Sasakawa Scientific Research Grant from The Japan Science Society.

## Conflict of interest

The authors declare that the research was conducted in the absence of any commercial or financial relationships that could be construed as a potential conflict of interest.

## Publisher’s note

All claims expressed in this article are solely those of the authors and do not necessarily represent those of their affiliated organizations, or those of the publisher, the editors and the reviewers. Any product that may be evaluated in this article, or claim that may be made by its manufacturer, is not guaranteed or endorsed by the publisher.
